# Supplementary material for: Heat adaptation of phage T7 under an extended genetic code
Source: Virus Evol. 2021 Dec 1;7(2):veab100. doi: 10.1093/ve/veab100 (PMC8923235; doi:10.1093/ve/veab100)
Supplement: veab100_Supp [file veab100_supp.zip › Sequencing_Analysis.zip!/Sequencing_Analysis/S3/evidence/JC_SIDE_2_51.html]

BRESEQ :: Evidence


| New junction evidence | | | | | | | | | | | |
| --- | --- | --- | --- | --- | --- | --- | --- | --- | --- | --- | --- |
|  | | seq id | position | reads (cov) | reads (cov) | score | skew | freq | annotation | gene | product |
| \* | ? | NC\_001604.1 | = 13586 | 2 (0.000) | 28 (0.020) | 14/592 | 38.4 | 65.1% | intergenic (–/–) | *–/–* | –/– |
|  | ? | NC\_001604.1 | 14356 = | 28 (0.020) |  |  |  |  | intergenic (–/–) | *–/–* | –/– |
| Rejected: Coverage evenness skew score above cutoff. | | | | | | | | | | | |

Only 100 of 1823 total aligned reads displayed.  

`GACAGACTGGCTGGAAGAAACCAGAAGTTACCAAGCTGAGTCATAAGGTGTTCGATAAGGACACTATGACCCACATCAAGGCTGGTGATTGGGTTAAGGTTGACATGGGAGTTGTTGGTGGATACGGCTACGTCCGCTCAGTTAGTGGCAAATATGCACAAGTGTCATACATCACAGGTGTTACTCCACGCGGTGCAATCGTTGCCGATAAGACCAACATGATTCACACAGGTTTCTTGACAGTTGTTTCATATGAAGAGATTGTTAAGTCACGATAATCAATAGGAGAAATCAATATGATCGTTTCTGACATCGAAGCTAACGCCCTCTTAGAGAGCGTCACTAAGTTCCACTGCGGGGTTATCTACGACTACTCCACCGCTGAGTACGTAAGCTACCGTCCGAGTGACTTCGGTGCGTATCTGGATGCGCTGGAAGCCGAGGTTGCACGAGGCGGTCTTATTGTGTTCCACAACGGTCACAAGTATGACGTTCCTGCATTGACCAAACTGGCAAAGTTGCAATTGAACCGAGAGTTCCACCTTCCTCGTGAGAACTGTATTGACACCCTTGTGTTGTCACGTTTGATTCATTCCAACC  >  NC_001604.1/14057‑14656`  

                                                                                                                                                                                                                                                                                                           `|`                                                                                                                                                                                                                                                                                                              
`gacgcctacgagaagtttcgctggagggtcccattctaatacgactcactaaaggagacacaccatgttcaaactgattaagaagttaggccaactgctggttcgtatgtacaacgtggaagccaagcgactgaacgatgaggctcgtaaagaggccacacagtcacgcgctctggcgattcgctccaacgaactggctgacagtgcatccactaaagttaccgaggctgcccgtgtggcaaaccaagctcaacagctttccaaattctttgagtaatcaaacaggagaaaTCAATATGAt                                                                                                                                                                                                                                                                                                             <  1:100087‑M2/10‑1 (MQ=255)`  
                      `ggagagtcccattctaatacgactcactaaaggagacacaccatgttcaaactgattaagaagttaggccaactgctggttcgtatgtacaacgtggaagccaagcgactgaacgatgaggctcgtaaagaggccacacagtcacgcgctctggcgattcgctccaacgaactggctgacagtgcatccactaaagttaccgaggctgcccgtgtggcaaaccaagctcaacagctttccaaattctttgagtaatcaaacaggagaaaCCATTATGATCGTTTCTGACATCGAAGCTAAc                                                                                                                                                                                                                                                                                       >  1:6747‑M2/270‑301 (MQ=255)`  
                           `gtcccattctaatacgactcactaaaggagacacaccttgttcaaactgattaagaagttaggccaactgctggttcgtatgtacaacgtggaagccaagcgactgaacgatgaggctcgtaaagaggccacacagtcacgcgctctggcgattcgctccaacgaactggctgacagtgcatccactaaagttaccgaggctgcccgtgtggcaaaccaagctcaacagctttccaaattctttgagtaatcaaacaggagaaaTCAATATGATCGTTTCTGACATCGAAGCTAACGCCct                                                                                                                                                                                                                                                                                  >  1:39764‑M2/265‑301 (MQ=255)`  
                           `gtcccattctaatacgactcactaaaggagacacaccatgttcaaactgattaagaagttaggccaactgctggttcgtatgtacaatgtggaagccaagcgactgaacgatgaggctcgtaaagaggccacacagtcacgcgctctggcgattcgctccaacgaactggctgacagtgcatccactaaagttaccgaggctgcccgtgtggcaaaccaagctcaacagctttccaaattctttgagtaatcaaacaggagaaaTCAATATGATCGTTTCTGACatcgaagctctgtctct                                                                                                                                                                                                                                                                                  >  1:104278‑M2/265‑293 (MQ=255)`  
                           `gtcccattctaatacgactcactaaaggagacacaccatgttcaaactgattaagaagttaggccaactgctggttcgtatgtacaacgtggaagccaagcgactgaacgatgaggctcgtaaagaggccacacagtcacgcgctctggcgattcgctccaacgaactggctgacagtgcatccactaaagttaccgaggctgcccgtgtggcaaaccaagctcaacagcgttccaaattctttgagtaatcaaacaggagaaaCAATTATGATCGTTTCTGACATCGAAGCTAAcgccat                                                                                                                                                                                                                                                                                  >  1:280875‑M2/265‑299 (MQ=255)`  
                            `tcccatactaatacgactcactaaaggagacacaccatgttcaaactgattaagaagttaggccaactgctggttcgtatgtacaacgtggaagccaagcgactgaacgatgaggctcgtaaagaggccacacagtcacgcgctctggcgattcgctccaacgaactggctgacagtgcatccactaaagttaccgaggctgcccgtgtggcaaaccaagctcaacagctttccaaattctttgagtaatcaaacaggagaaaTCAATATGATCGTTTCTGACATCGAAGCTAACGCCctc                                                                                                                                                                                                                                                                                 <  1:190558‑M2/38‑1 (MQ=255)`  
                                `attctaatacgactcactaaaggagacacaccatgttcaaactgattaagaagttaggccaactgctggttcgtatgtacaacgtggaagccaagcgactgaacgatgaggctcgtaaagaggccacacagtcacgcgctctggcgattcgctccaacgaactggctgacagtgcatccactaaagttaccgaggctgcccgtgtggcaaaccaagctcaacagctttccaaattctttgagtaatcaaacaggagaaaTCAATATGATCGTTTCTGACATCGAAGCTAACGCCCTCTTag                                                                                                                                                                                                                                                                             >  1:86229‑M2/260‑301 (MQ=255)`  
                                 `ttctaatacgactcactaaaggagacacaccatgttcaaactgattaagaagttaggccaactgctggttcgtatgtacaacgtggaagccaagcgactgaacgatgaggctcgtaaagaggccacacagtcacgcgctctggcgattcgctccaacgaactggctgacagtgcatccactaaagttaccgaggctgcccgtgtggcaaaccaagctctacagctttccaaattctttgagtaatcaaacaggagaaaTCAATATGATCGTTTCTGACATCGAAGCTAACGCCCTCTTaga                                                                                                                                                                                                                                                                            >  1:309536‑M2/259‑301 (MQ=255)`  
                                 `ttctaatacgactcactaaaggagacacaccatgttcaaactgattaagaagttaggccaactgctggttcgtatgtacaacgtggaagccaagcgactgaacgatgaggctcgtaaagaggccacacagtcacgcgctctggcgattcgctccaacgaactggctgacagtgcatccactaaagttaccgaggctgcccgtgtggcaaaccaagctcaacagctttccaaattctttgagtaatcaaacaggagaaaCCATTATGATCGTTTCTGACATCGAAGCTAACGCCCTCTTaga                                                                                                                                                                                                                                                                            >  1:109457‑M2/259‑301 (MQ=255)`  
                                 `ttctaatacgactcactaaaggagacacaccatgttcaaactgattaagaagttaggccaactgctggttcgtatgtacaacgaggatgccaagcgactgaacgatgaggctcgtcaagaggccacaatgtcacgcgctctggcgattcgctcccacgaactggctgacagtgcatccactaaagttaccgagtctgcccgtgtggcaaaccaagatccacagctatccaaattctttgagtaatcgaacacgagaaaTCAATATGATCGTTTCTTACAGCTAAGCTAACGACctctttgc                                                                                                                                                                                                                                                                            >  1:44010‑M2/259‑298 (MQ=255)`  
                                 `ttctaatacgactcactaaaggagacacaccatgttcaaactgattaagaagttaggccaactgctggttcgtatgtacaacatggaagccaagcgactgaacgatgaggctcgtaaagaggccacacagtcacgcgctctggcgattcgctccaacgaactggctgacagtgcatcccctaaagttaccgagtctgcccgtttggccaaccaagctcaacagctttccaaattctttgagtaatcaaacaggagaaaCCATTATGCTCGGTTCTGACATCGACGATCACGACCTATTaga                                                                                                                                                                                                                                                                            >  1:26982‑M2/259‑301 (MQ=255)`  
                                  `tctaatacgactcactaaaggagacacaccatgttcaaactgattaagaagttaggccaactgctggttcgtatgtacaacgtggaagccaagcgactgaacgatgaggctcgtaaagaggccacacagtcacgcgctctggcgattcgctccaacgaactggctgacagtgcatccactaaagttaccgaggctgcccgtgtggcaaaccaagctcaacagctttccaaattctttgagtaatcaaacaggagaaaTCAATATGATCGTTTCTGACATCGAAGCTAACGCCCTCTTagag                                                                                                                                                                                                                                                                           <  1:172565‑M2/44‑1 (MQ=255)`  
                                  `tctaatacgactcactaaaggagacacaccatgttcaaactgattaagaagttaggccaactgctggttcgtatgtacaacgtggaagccaagcgactgaacgatgaggctcgtaaagaggccacacagtcacgcgctctggcgattcgctccaacgaactggctgacagtgcatccactaaagttaccgaggctgcccgtgtggcaaaccaagctcaacagctttccaaattctttgagtaatcaaacaggagaaaTCAATATGATCGTTTCTGACATCGAAGCTAACGCCCTCTTagag                                                                                                                                                                                                                                                                           <  1:49591‑M2/44‑1 (MQ=255)`  
                                  `tctaatacgactcactaaaggagacacaccatgttcaaactgattaagaagttaggccaactgctggttcgtatgtacaacgtggaagccaagcgactgaacgatgaggctcgtaaagaggccacacagtcacgcgctctggcgattcgctccaacgaactggctgacagtgcatccactaaagttaccgaggctgcccgtgtggcaaaccaagctcaacagctttccaaattctttgagtaatcaaacaggagaaaTCAATATGATCGTTTCTGACATCGAAGCTAACGCCCTATTagag                                                                                                                                                                                                                                                                           <  1:53915‑M2/44‑1 (MQ=255)`  
                                  `tctaatacgactcacgaaaggagaaacaccaagtacaaactgattgagaaattaggccaactgctagttcgtaagtacaacgtggaagccaagcgactgaacgatgaggctcgtaaataggccacacaatcgcgctctctggcaattcgctccaacgaacaggctgacagtgcatccactaaaggtaccgaggctggccgtgtgacaaaccaagctcaacagctttccaaattctttgagtaatcaaacaggagaaaTCAATATGATCGTTTCTGACATCGAAGCTAACGCCCTCTTagag                                                                                                                                                                                                                                                                           <  1:215788‑M2/44‑1 (MQ=255)`  
                                  `gagatgtgtataagagacaggagacacaccatgttcaaactgattaagaagttaggccaactgctggttcgtatgtacaacgtggaagccaagcgactgaacgatgaggctcgtaaagaggccacacagtcacgcgctctggcgattcgctccaacgaactggctgacagtgcatccactaaagttaccgaggctgcccgtgtggcaaaccaagctcaacagctttccaaattctttgagtaatcaaacaggagaaaTCAATATGATCGTTTCTGACATCGAAGCTAACGCCCTCTTagag                                                                                                                                                                                                                                                                           <  1:112839‑M2/44‑1 (MQ=255)`  
                                   `ctaatacgactcactaaaggagacacaccatgttcaaactgattaagaagttaggccaactgctggttcgtatttacaacgtggaagccaagcgactgaacgatgaggctcgtaaagaggccacacagtcacgcgctctggcgattcgctccaacgaactggctgacagtgcattcactaaagttaccgaggctgcccgtgtggcaaaccaagctcaacagctttccaaattctttgagtactcaaacaggagaaaCCATTATGATCGTTTCTGACATCGAAGCTAACCCCCTCTTagaga                                                                                                                                                                                                                                                                          >  1:151151‑M2/257‑301 (MQ=255)`  
                                   `ctaatacgactcactaaaggagacacaccatgttcaaactgattaagaagttaggccaactgctggttcgtatgtacaacgtggaagccaagcgactgaacgatgaggctcgtaaagaggccacacagtcacgcgctctggcgattcgctccaacgaactggctgacagtgcatccactaaagttaccgaggctgcccgtgtggcaaaccaagctcaacagctttccaaattctttgagtaatcaaacaggagaaaTCAATATGATCGTTTCTGACATCGACGCTAACGCCCTCTtagagc                                                                                                                                                                                                                                                                          >  1:57004‑M2/257‑300 (MQ=255)`  
                                    `taatacgactcactaaaggagacacaccatgttcaaactgattaagaagttaggccaactgctggttcgtatgtacaacgtggaagccaagcgactgaacgatgaggctcgtaaagaggccacacagtcacgcgctctggcgattcgctccaacgaactggctgacagtgcatccactaaagttaccgaggctgcccgtgtggcaaaccaagctcaacagctttccaaattctttgagtaatcaaacaggagaaaTCAATATGATCGTTTCTGACATCGAAgctaacgccctgtctcttat                                                                                                                                                                                                                                                                         >  1:337866‑M2/256‑292 (MQ=255)`  
                                    `gtgggctcggagatgtgtataagagacagtgttcaaactgattaagaagttaggccaactgctggttcgtatgtacaacgtggaagccaagcgactgaacgatgaggctcgtaaagaggccacacagtcacgcgctctggcgattcgctccaacgaactggctgacagtgcatccactaaagttaccgaggctgcccgtgtggcaaaccaagctcaacagctttccaaattctttgagtaatcaaacaggagaaaTCAATATGATCGTTTCTGACATCGAAGCTAACGCCCTCTTagagag                                                                                                                                                                                                                                                                         <  1:135824‑M2/46‑1 (MQ=255)`  
                                    `aaatacgactcactaaaggagacacaccatgttcaaactgattaagaagttaggccaacttctggttcgtatgtacaacgtggaagccaagcgactgaacgatgaggctcgtaaagaggccacacagtcacgcgctctggcgattcgctccaacgaactggctgacagtgcatccactaaagttaccgaggctgcccgtgtggcaaaccaagctcaacagctttccaaattctttgagtaatcaaacaggagaaaCCATTATGATCGTTTCTGACATCGAAGCTAACGCCCTCTTagagag                                                                                                                                                                                                                                                                         <  1:345411‑M2/46‑1 (MQ=255)`  
                                     `aatacgactcactaaaggagacacaccatgttcaaactgattaagaagttaggccaactgctggttcgtatgtacaacgtggaagccaagcgactgaacgatgaggctcgtaaagaggccacacagtcacgcgctctggcgattcgctccaacgaactggctgacagtgcatccactaaagttaccgaggctgcccgtgtggcaaaccaagctcaacagctttccaaattctttgagtaatcaaacaggagaaaCCATTATGATCGTTTCTGACATCGAAGCTAACGCCCTCTTAGAGAGc                                                                                                                                                                                                                                                                        >  1:208327‑M2/255‑301 (MQ=255)`  
                                            `ctcactaaaggagacacaccatgttcaaactgattaagaagttaggccaactgctggttcgtatgtacaacgtggaagccaagcgactgaacgatgaggctcgtaaagaggccacacagtcacgcgctctggcgattcgctccaacgaactggctgacagtgcatccactaaagttaccgaggctgcccgtgtggcaaaccaagctcaacagctttccacattctttgagtaatcaaacaggagaaaTCAATATGATCGTTTCTGACATCGAAGCTAacgccctcttagagctgtctcttt                                                                                                                                                                                                                                                                 >  1:325103‑M2/248‑291 (MQ=255)`  
                                              `cactaaaggatacacaccatgttcaaactgattaagaagttaggccaactgctggttcgtatgtacaacgtggaagccaagcgactgaacgatgaggctcgtaaagaggccacacagtcacgcgctctggcgattcgctccaacgaactggctgacagtgcatccactaaagttaccgaggctgcccgtgtggcaaaccaagctcaacagctttccaaattctttgagtaatcaaacaggagaaaTCAATATGATCGTTTCTGACATCGAAACTAACGCCCTCTTAGAGAGCGTCACtaat                                                                                                                                                                                                                                                               >  1:253539‑M2/246‑300 (MQ=255)`  
                                              `cactaaaggagacacaccatgttcaaactgattaagaagttaggccaactgctggttcgtatgtacaacgtggaagccaagcgactgaacgatgaggctcgtaaagaggccacacagtcacgcgctctggcgattcgctccaacgaactggctgacagtgcatccactaaagttaccgaggctgcccgtgtggcaaaccaagctcgacagctttccaaattctttgagtaatcaaacaggagaaaTCAATATGATCGATTCTGACATCGAAGCtaacgccctcttagagctgtctcttata                                                                                                                                                                                                                                                               >  1:287510‑M2/246‑289 (MQ=255)`  
                                              `cactaaaggagacacaccatgttcaaactgattaagaagttaggccaactgctggttcgtatgtacaacgtggaagccaagcgactgaacgatgaggctcgtaaagaggccacacagtcacgcgctctggcgattcgctccaacgaactggctgacagtgcatccactaaagttaccgaggctgcccgtgtggcaaaccaagctcaacagctttccaaattctttgagtaatcaaacaggagaaaTCAATATGATCGTTTCTGACATCGAAGCTAACGCCCTCTTAGAGAGCGTCACTAAg                                                                                                                                                                                                                                                               >  1:147659‑M2/246‑301 (MQ=255)`  
                                              `cactaaaggagacacaccatgttcaaactgattaagaagttaggccaactgctggttcgtatgtacaacgtggaagccaagcgactgaacgatgaggctcgtaaagaggccacacagtcacgcgctctggcgattcgctccaacgaactggctgacagtgcatccactaaagttaccgaggctgcccgtgtggcaaaccaagctcaacagctttccaaattctttgagtaatcaaacaggagaaaTCAATATGATCGTTTCTGACATCGAAGCTAACGCCCTCTTAGAGAGCGTCACTAAg                                                                                                                                                                                                                                                               >  1:138954‑M2/246‑301 (MQ=255)`  
                                              `cactaaaggagacacaccatgttcaaactgattaagaagttaggccaactgctggttcgtatgtacaacgtggaagccaagcgactgaacgatgaggctcgtaaagaggccacacagtcacgcgctctggcgattcgctccaacgaactggctgacagtgcatccactaaagttaccgaggctgaccgtgtggcaaaccaagctcaacagctttccaaattctttgagtaatcaaacaggagaaaCCATTATGATCGTTTCTGACATCGAAGCtaacgccctcttagagctgtctcttata                                                                                                                                                                                                                                                               >  1:7367‑M2/246‑289 (MQ=255)`  
                                                `ctaaaggagacacaccatgttcaaactgattaagaagttaggccaactgctggttcgtatgtacaacgtggaagccaagcgactgaacgatgaggctcgtaaagaggccacacagtcacgcgctctggcgattcgctccaacgaactggctgacagtgcatccactaaagttaccgaggctgcccgtgtggcaaaccaagctcaacagctttccaaattctttgagtaatcaaacaggagaaaTCAATATGATCGTTTCTGACATCAAAGCTAACGCCCTCTTAGAGCGCTTCACTAAGtt                                                                                                                                                                                                                                                             >  1:212552‑M2/244‑301 (MQ=255)`  
                                                     `ggagacacaccatgttcaaactgattaagaagttaggccaactgctggttcgtatgtacaacgtggaagccaagcgactgaacgatgaggctcgtaaagaggccacacagtcacgcgctctggcgattcgctccaacgaactggctgacagtgcatccactaaagttaccgaggctgcccgtgtggcaaaccacgctctacagctttccaaattctttgagtaatcaaacaggagaaaTCAATATGATCGTTTCTGACATCGAAGCTAacgccctcttagagagctgtctcttatacacat                                                                                                                                                                                                                                                        >  1:70636‑M2/239‑285 (MQ=255)`  
                                                     `ggagacacaccatgttcaaactgattaagaagttaggccaactgctagttcgtatgtacaacgtggaagccaagcgactgaacgatgaggctcgtaaagaggccacacagtcacgcgctctggcgattcgctccaacgaactggctgacagtgcatccactaaagttaccgaggctgcccgtgtggcaaaccaagctcaacagctttccaaattctttgagtaatcaaacaggagaaaTCAATATGATCGTTTCTGACATCGAAGCTAACGCCCTCTTAGAGAGCGTCACTAAGTTCCacc                                                                                                                                                                                                                                                        >  1:328425‑M2/239‑300 (MQ=255)`  
                                                      `gagacacaccatgttcaaactgattaagaagttaggccaactgctggttcgtatgtacaacgtggaagccaagcgactgaacgatgaggctcgtaaagaggccacacagtcacgcgctctggcgattcgctccaacgaactggctgacagtgcatccactaaagttaccgaggctgcccgtgtggcaaaccaagctcaacagctttccaaattctttgagtaatcaaacaggagaaaTCAATATGATCGTTTCTGACATCGAAGCTAACGCCCTCTTAGAGAGCGTCACTAAGTTCCActc                                                                                                                                                                                                                                                       >  1:204982‑M2/238‑300 (MQ=255)`  
                                                      `gagacacaccatgttcaaactgattaagaagttaggccaactgctggttcgtatgtacaacgtggaagccaagcgactgaacgatgaggctcgtaaagaggccacacagtcacgcgctctggcgattcgctccaacgaactggctgacagtgcatccactaaagttaccgaggctgcccgtgtggcaaaccaagctcaacagctttccaaattctttgagtaatcaaacaggagaaaTCAATATGATCGTTTCTGACATCGAAGCTAACGCCCTCTTAGAGAGCGTCACTAAGTTCCACTg                                                                                                                                                                                                                                                       <  1:167100‑M2/64‑1 (MQ=255)`  
                                                            `aaccatgttcaaactgattaaaaagttgagccaaacgctggttcgtatttaccacatggaagccaagcgaatgaacgatgaggctcgtaaagaggccacacagtcacgcgctctggcgattcgctccaactaactggctgacagtgcatccactaaagttaccgaggctgcccgtgtggcaaaccaagctcaacagatttccaaattctttgagtaatcaaacaggagaaaTCAATATGATCGTTTCTGACATCGAAGCTAACGCCCTCTTAGAGAGCGTCACTAAGTTCCACTGCGGGGt                                                                                                                                                                                                                                                 <  1:91627‑M2/70‑1 (MQ=255)`  
                                                              `ccatgttcaaactgattaagaagttaggccaactgctggttcgtatgtacaacgtggaagccaagcgactgaacgatgaggctcgtaaagaggccacacagtcacgcgctctggcgattcgctccaacgaactggctgacagtgcatccactaaagttaccgaggctgcccgtgtggcaaaccaagctcaacagctttccaaattctttgagtaatcaaacaggagaaaTCAATATGATCGTTTCTGACATCGAAGCTAACGCCCTCTTAGAGAGCGTCACTAAGTTCCACTGCGGGGTTa                                                                                                                                                                                                                                               <  1:29165‑M2/72‑1 (MQ=255)`  
                                                                 `tgttcaaactgattaagaagttaggccaactgctggttcgtatgtacaacgtgtaagccaagcgactgaacgatgaggctcgtaaagaggccacacagtcacgcgctctggcgattcgctccaacgaactggctgacagtgcatccactaaagttaccgaggctgcccgtgtggcaaaccaagctcaacagctttccaaattctttgagtaatcaaacaggagaaaTCAATATGATCGTTTCTGACATCGAAGCTAACGCCCTCTTAGAGAGCGTCACTACGTTCCACTGCGGGGttctcc                                                                                                                                                                                                                                            >  1:27226‑M2/227‑300 (MQ=255)`  
                                                                 `tgttcaaactgattaagaagttaggccaactgctggttcgtatgtacaacgtggaagccaagcgactgaacgatgaggctcgtaaagaggccacacagtcacgcgctctggcgattcgctccaacgaactggctgacagtgcatccactaaagttaccgaggctgcccgtgtggcaaaccaagctcaacagctttccaaattctttgagtaatcaaacaggagaaaCCATTATGATCGTTTCTGACATCGAAGCTAACGCCCTCTTAGAGAGCGTCACTAAGTTCCACTGCGGGGTTATCt                                                                                                                                                                                                                                            <  1:289726‑M2/75‑1 (MQ=255)`  
                                                                 `ggttcaaattgattaagaagttaggccaacggctggttcgtatgtacaacgtggaagccaagcgactgaacgatgaggctcgtaaagaggccacacagtcacgcgctctggcgattcgctccaacgaactggctgacagtgcatccactaaagttaccgaggctgcccgtgtggcaaaccaagctcaacagctttccaaattctttgagtaatcaaacaggagaaaTCAATATGATCGTTTCTGACATCGAAGCTAACGCCCTCTTAGAGAGCGTCACTAAGTTCCACTGCGGGGTTATCt                                                                                                                                                                                                                                            <  1:318718‑M2/75‑1 (MQ=255)`  
                                                                 `aaagagacaggattaagaagttaggcccactgctggttcgtatgtacaacgtggaagccaagcgactgaacgatgaggctcgtaaagaggccacacagtcacgcgctctggcgattcgctccaacgaactggctgacagtgcagccactaaagttaccgaggctgcccgtgtggcaaaccatgctcaacagctttccaaattctttgagtaatcaaaccggagaaaTCAATATGATCGTTTCTGACATCGAAGCTAACGCCCTCTTAGAGAGCGTCACTAAGTTCCACTGCGGGGTTATCt                                                                                                                                                                                                                                            <  1:340545‑M2/75‑1 (MQ=255)`  
                                                                  `gttcaaactgattaagaagttaggccaactgctggttcgtatgtacaacgtggaagccaagcgactgaacgatgaggctcgtaaagaggccacacagtcacgcgctctggcgattcgctccaacgaactggctgacagtgcatccactaaagttaccgaggctgcccgtgtggcaaaccaagctcaacagctttccaaattctttgagtaatcaaacaggagaaaTCAATATGATCGTTTCTGACATCGAAGCTAACGCCCTCTTAGAGAGCGTCACTAAGTTCCACTGCGGGGTTATCTa                                                                                                                                                                                                                                           >  1:242640‑M2/226‑301 (MQ=255)`  
                                                                      `aaaccgattaagaagttaggccaactgctggttcgtatgtacaacgtggaagccaagcgactgaacgatgaggctcgtaaagaagccacacagtcacgcgctctggcgattcgctccaacgaactggctgacagtgcatccactaaagttaccgaggctgcccgtgtggcaaaccaagctcaacagctttccaaattctttgagtaatcaaacaggagaaaTCAATATGATCGTTTCTGACATCGAAGCTAACGCCCTCTTAGAGAGCGTCACTAAGTTCCACTGCGGGGTTATCTACGac                                                                                                                                                                                                                                       <  1:117918‑M2/80‑1 (MQ=255)`  
                                                                           `gattaagaagttaggccaactgctggttcgtatgtacaacgtggaagccaagcgactgaacgatgaggctcgtaaagaggccacacagtcacgcgctctggcgattcgctccaacgaactggctgacagtgcatccactaaagttaccgaggctgcccgtgtggcaaaccaagctcaacagctttccaaattctttgagtaatcaaacaggagaaaTCAATATGATCGTTTCTGACATCGAAGCTAACGCCCTCTTAGAGAGCGTCACTAAGTTCCACTGCGGGGTTATCTACGActcctc                                                                                                                                                                                                                                  >  1:335182‑M2/217‑301 (MQ=255)`  
                                                                            `attaagaagttaggccaactgctggttcgtatgtacaacgtggaagccaagcgactgaacgatgaggctcgtaaagaggccacacagtcacgcgctctggcgattcgctccaacgaactggctgacagtgcatccactaaagttaccgaggctgcccgtgtggcaaaccaagctcaacagctttccaaattctttgagtaatcaaacaggagaaaTCAATATGATCGTTTCTGACATCGAAGCTAACGCCCTCTTAGAGAGCGTCACTAAGTTCCACTgcggggttatctctgtcacttat                                                                                                                                                                                                                                 >  1:63532‑M2/216‑290 (MQ=255)`  
                                                                            `attaagaagttaggccaactgctggttcgtatgtacaacgtggaagccaagcgactgaacgatgaggctcgtaaagaggccacacagtcacgcgctctggcgattcgctccaacgaactggctgacagtgcatccactaaagttaccgaggctgcccgtgtggcaaaccaagctcaacagctttccaaattctttgagtaatcaaacaggagaaaTCAATATGATCGTTTCTGACATCGAAGCTAACGCCCTCTTAGAGAGCGTCACTAAGTTCCACTGCGGGGTTATCTACGACTACTcc                                                                                                                                                                                                                                 >  1:278362‑M2/216‑301 (MQ=255)`  
                                                                              `aaagagacagggccaactgctggttcgtatgtacaacgtggaagccaagcgactgaacgatgaggctcgtaaagaggccacacagtcacgcgctctggcgattcgctccaacgaactggctgacagtgcatccactaaagttaccgaggctgcccgtgtggcaaaccaagctcaacagctttccaatttctttgagtaatcaaacaggagaaaTCAATATGATCGTTTCTGACATCGAAGCTAACGCCCTCTTAGAGAGCGTCACTAAGTTCCACTGCGGGGTTATCTACGACTACTCCAc                                                                                                                                                                                                                               <  1:37738‑M2/88‑1 (MQ=255)`  
                                                                                   `agttaggccaactgctggttcgtatgtacaacgtggaagccaagcgactgaacgatgaggctcgtaaagaggccacacagtcacgcgctctggcgattcgctccaacgaactggctgacagtgcatccactaaagttaccgaggctgcccgtgtggcaaaccaagctcaacagctttccaaattctttgagtaatcaaacaggagaaaTCAATATGATCGTTTCTGACATCGAAGCTAACGCCCTCTTAGAGAGCGTCACTAAGTTCCACTGCGGGGTTATCTACGACTACTCCACCGCTg                                                                                                                                                                                                                          <  1:260305‑M2/93‑1 (MQ=255)`  
                                                                                           `caactgctggttcgtatgtacaacggggaagccaagcgactgaacgatgaggctcgtaaagaggccacacagtcacgcactctggcgattcgctccaacgaactggctgacagtgcatccactaaagttaccgaggctgcccgtgtggcaaaccaagctcaacagctttccaaattctttgagtaatcaaacaggagaaaCCATTATGATCGTTTCTGACATCGAAGCTAACGTCCTCTTAGAGAGAGTCACTAAGTTCCACTGCGGGGTTATCTACGACTACTCCACCGCTGAGTACGTa                                                                                                                                                                                                                  <  1:128303‑M2/101‑1 (MQ=255)`  
                                                                                             `actgctggttcgtatgcacaacgtggaagccaagcgactgaacgatgaggctcgtaaagaggccacacagtcacgcgctctggcgattcgctccaacgaactggctgacagtgcatccactaaagttaccgaggctgcccgtgtggcaaaccaagctcaacagctttccaaattctttgagtaatcaaacaggagaaaTCAATATGATCGTTTCTGACATCGAAGCTAACGCCCTCTTAGAGAGCGTCACTAAGTTCCACTGCGGGGTTATCTACGACTACTCCACCGCTGAGTACGTAAg                                                                                                                                                                                                                <  1:312441‑M2/103‑1 (MQ=255)`  
                                                                                                    `gttcgtatgtacaacgtggaagccaagcgactgaacgatgaggctcgtaaagaggccacacagtcacgcgctctggcgattcgctccaacgaactggctgacagtgcatccactaaagttaccgaggctgcccgtgtggcaaaccaagctcaacagctttccaaattctttgagtaatcaaacaggagaaaTCAATATGATCGTTTCTGACATCGAAGCTAACGCCCTCTTAGAGAGCGTCACTAAGTTCCACTGCGGGGTTATCTACGACTACTCCACCGCTGAGTACGTAAGCTACcgc                                                                                                                                                                                                         >  1:80299‑M2/192‑300 (MQ=255)`  
                                                                                                     `ttcgtatgtacaacgtggaagccaagcgactgaacgatgaggctcgtaaagaggccacacagtcacgcgctctggcgattcgctccaacgaactggctgacagtgcatccactaaagttaccgaggctgcccgtgtggcaaaccaagctcaacagctttccaaattctttgagtaatcaaacaggagaaaTCAATATGATCGTTTCTGACATCGAAGCTAACGCCCTCTTAGAGAGCGTCACTAAGTTCCACTGCGGGGTTATCTACGACTACTCCACCGCTGAGTACGTAAGCTACCGTc                                                                                                                                                                                                        <  1:233098‑M2/111‑1 (MQ=255)`  
                                                                                                     `ttcgtatgtacaacgtggaagccaagcgactgaacgatgaggcacgtaaagaggccacacagtcacgcgctctggcgattcgctccaacgaactggctgacagtgcatccactaaagttaccgaggctgcccgtgtggcaaaccaagctcaacagctttccaaattctttgagtaatcaaacaggagaaaTCAATATGATCGTTTCTGACATCGAAGCTAACGCCCTCTTAGAGAGCGTCACTAAGTTCCACTGCGGGGTTATCTACGACTACTCCACCGCTGAGTACGTAAGCTACCGTc                                                                                                                                                                                                        <  1:163837‑M2/111‑1 (MQ=255)`  
                                                                                                                 `acgtggaagccaagcgactgaacgatgaggctcgtaaagaggccacacagtcacgcgctctggcgattcgctccaacgaactggctgacagtgcatccactaaagttaccgaggctgcccgtgtggcaaaccaagctcaacagctttccaaattctttgagtaatcaaacaggagaaaTCAATATGATCGTTTCTGACATCGAAGCTAACGCCCTCTTAGAGAGCGTCACTAAGTTCCACTGCGGGGTTATCTACGACTACTCCACCGCTGAGTACGTAAGCTACCGTCCGAGTGACTTCg                                                                                                                                                                                            >  1:226123‑M2/179‑301 (MQ=255)`  
                                                                                                                  `cgtggaagccaagcgactgaacgatgaggctcgtaaagaggccacacagtcacgcgctctggcgattcgctccaacgaactggctgacagtgcatccactaaagttaccgaggctgcccgtgtggcaaaccaagctcaacagctttccaaattctttgagtaatcaaacaggagaaaTCAATATGATCGTTTCTGACATCGAAGCTAACGCCCTCTTAGAGAGCGTCACTAAGTTCCACTGCGGGGTTATCTACGACTACTCCACCGCTGAGTACGTAAGCTACCGTCCGAGTGACTTCgg                                                                                                                                                                                           >  1:199862‑M2/178‑301 (MQ=255)`  
                                                                                                                     `ggaagccaagcgactgaacgatgaggctcgtaaagaggccacacagtcacgcgctctggcgattcgctccaacgaactggttgacagtgcatccactaaagttaccgaggctgcccgtgtggcaaaccaagctcaacagctttccaaattctttgagtaatcaaacaggagaaaTCAATATGATCGTTTCTGACATCGAAGCTAACGCCCTCTTAGAGAGCGTCACTAAGTTCCACTGCGGGGTTATCTACGACTACTCCACCGCTGAGTACGTAAGCTACCGTCCGAGTGACTTCGGTGc                                                                                                                                                                                        <  1:115450‑M2/127‑1 (MQ=255)`  
                                                                                                                      `gaagccaagcgactgaacgatgaggctcgtaaagaggccacacagtcacgcgctctggcgattcgatcctacgaactggctgacagtgcatccactaaagttaccgaggctgcccgtgtggcaaaccaagctcaacagctgtccaaattctttgagtaatcaaacaggagaaaTCAATATGATCGTTTCTGACATCGAAGCTAACGCCCTCTTAGAGAGCGTCACTCAGTTCCACTGCGGGGTTATCTACGACTACTCCACCGCTGAGTACGTAAGCTACCGTCCGAGTGACTTCGGTGCg                                                                                                                                                                                       <  1:130976‑M2/128‑1 (MQ=255)`  
                                                                                                                          `ccaagcgactgaacgatgaggctcgtaaagaggccacacagtcacgcgctctggcgattcgctccaacgaactggctgacagtgcatccactaaagttaccgaggctgcccgtgtggcaaaccaagctcaacagctttccaaattctttgagtaatcaaacaggagaaaTCAATATGATCGTTTCTGACATCGAAGCTAACGCCCTCTTAGAGAGCGTCACTAAGTTCCACTGCGGGGTTATCTACGACTACTCCACCGCTGAGTACGTAAGCTACCGTCCGAGTGACTTCGGTGCGTATc                                                                                                                                                                                   <  1:247491‑M2/132‑1 (MQ=255)`  
                                                                                                                           `caagcgacggaacgatgaggatcgtaaagaggccacacagtcacgcgctcgggcgattcgctccaacgaactggctgacagtgcatccactaaagttaccgaggctgcccgtgtggcaaacaaagctcaacagctgtccaaattctttgagtaatcaaacaggagaaaCCATTATGATCGTTTCTGACATCGAAGCTAACGCCCTCTTAGAGAGCGTCACTAAGTTCCACTGCGGGGTTATCTACGACTACTCCACCGCTGAGTACGTAAGCTACCGTCCGAGTGACTTCGGTGCGTATCt                                                                                                                                                                                  <  1:230552‑M2/133‑1 (MQ=255)`  
                                                                                                                              `gcgactgaacgatgaggctcgtaaagaggccacacagtcacgcgctctggcgattcgctccaacgaactggctgacagtgcatccactaaagttaccgaggctgcccgtgtggcaaaccaagctcaacagctttccaaattctttgagtaatcaaacaggagaaaTCAATATGATCGTTTCTGACATCGAAGCTAACGCCCTCTTAGAGAGCGTCACTAAGTTCCACTGCGGGGTTATCTACGACTACTCCACCGCTGAGTACGTAAGCTACCGTCCGAGTGACTTCGGTGCGTATCTGGa                                                                                                                                                                               >  1:12047‑M2/166‑301 (MQ=255)`  
                                                                                                                              `gcgactgaacgatgaggctcgtaaagaggccacacagtcacgcgctctggcgattcgctccaacgaactggctgacagtgcatccactaaagttaccgaggctgcccgtgtggcaaaccaagctcaacagctttccaaattctttgagtaatcaaacaggagaaaTCAATATGATCGTTTCTGACATCGAAGCTAACGCCCTCTTAGAGAGCGTCACTAAGTTCCACTGCGGGGTTATCTACGACTACTCCACCGCTGAGTACGTAAGCTACCGTCCGAGTGACTTCGGTGCGTATCTGGa                                                                                                                                                                               <  1:223174‑M2/136‑1 (MQ=255)`  
                                                                                                                               `cgactgaacgatgaggctcgtaaagacgccacacagtcacgcgctctgtcgattcgctccaacgaactggctgacagtgcatccactaaagttaccgaggctgcccgtgtggcaaaccaagctcaacagctttccaaattctttgagtaatcaaacaggagaaaTCAATATGATCGTTTCTGACATCGAAGCTAACGCCCTCTTAGAGAGCGTCACTAAGTTCCACTGCGGGGTTATCTACGACTACTCCACCGCTGAGTACATAAGCTACCGTCCGAGTGACTTCGGTGCGTATCTGGAt                                                                                                                                                                              <  1:83216‑M2/137‑1 (MQ=255)`  
                                                                                                                                                    `aaagaggccacacagtcacgcgctctggcgatgcgctccaacgaactggctgacagtgcatccactaaagttaccgagtctgcccgtgtggcaaaccaagctcaacagctttccaaattctttgagtaatcaaacaggagaaaTCAATATGATCGTTTCTGACATCGAAGCTAACGCCCTCTTAGAGAGCGTCACTAAGTTCCACAGCGGGGTTATCTACGACTACTCCACCGCTGAGTACGTAAGCTACCGTCCGAGTGACTTCGGTGCGTATCTGGATGCGCTGGAAGCCGAGGTTGCa                                                                                                                                                         <  1:175669‑M2/158‑1 (MQ=255)`  
                                                                                                                                                     `atgaggtcacacatgcacgtgctctcacggttcgtgcaaacgaacttgctgacattgcatccactaaagttaccgaggctgcccgtgtgtcaaaccaagctcaacagctttccaaattctttgagtaatcaaacaggagaaaCCATTATGATCGTTTCTGACATCGAAGCTATCGCCCTCTTAGAGAGCGTCACTAAGTTCCACTGCGTGGTTATCTACGACTACTCCACCGCTGAGTACGTAAGCTACCGTCCGAGTGACTTCGGTGCGTATCTGGATGCGCTGGAAGCCGAGGTTGCAc                                                                                                                                                        <  1:158353‑M2/159‑1 (MQ=255)`  
                                                                                                                                                       `gaggccacacagtcacgcgctctggcgattcgctccaacgaactggctgacagtgcatccactaaagttaccgaggctgcccgtgtggcaaaccaagctcaacagctttccaaattctttgagtaatcaaacaggagaaaTCAATATGATCGTTTCTGACATCGAAGCTAACGCCCTCTTAGAGAGCGTCACTAAGTTCCACTGCGGGGTTATCTACGACTACTCCACCGCTGAGTACGTAAGCTACCGTCCGAGTGACTTCGGTGCGTATCTGGATGCGCTGGAAGCCGAGGTTGCAcgg                                                                                                                                                      >  1:300704‑M2/141‑300 (MQ=255)`  
                                                                                                                                                       `gaggccacacagtcacgcgctctggcgattcgctccaacgaactggctgacagtgcatccactaaagttaccgaggctgcccgtgtggcaaaccaagctcaacagctttccaaattctttgagtaatcaaacaggagaaaTCAATATGATCGTTTCTGACATCGAAGCTAACGCCCTCTTAGAGAGCGTCACTAAGTTCCACTGCGGGGTTATCTACGACTACTCCACCGCTGAGTACGTAAGCTACCGTCCGAGTGACTTCGGTGCGTATCTGGATGCGCTGGAAGCCGAGGTTGCACGa                                                                                                                                                      <  1:321395‑M2/161‑1 (MQ=255)`  
                                                                                                                                                       `gaggccacacagtcacgcgctctggcgattcgctccaacgaactggctgacagtgcatccactaaagttaccgaggctgcccgtgtggcaaaccaagctcaacagctttccaaattctttgagtaatcaaacaggagaaaTCAATATGATCGTTTCTGACATCGAAGCTAACGCCCTCTTAGAGAGCGTCACTAAGTTCCACTGCGGGGTTATCTACGACTACTCCACCGCTGAGAACGTAAGCTACCGTCCGAGTGACTTCGGTGCGTATCTGGATGCGCTGGAAGCCGAGGTTGCACGa                                                                                                                                                      >  1:28421‑M2/141‑301 (MQ=255)`  
                                                                                                                                                       `agatgtgtataagagacagctctggcgattcgctccaacgaactggctgacagtgcatccactaaagttaccgaggctgcccgtgtggcaaaccaagctcaacagctttccaaattctttgagtaatcaaacaggagaaaTCAATATGATCGTTTCTGACATCGAAGCTAACGCCCTCTTAGAGAGCGTCACTAAGTTCCACTGCGGGGTTATCTACGACTACTCCACCGCTGAGTACGTAAGCTACCGTCCGAGTGACTTCGGTGCGTATCTGGATGCGCTGGAAGCCGAGGTTGCACGa                                                                                                                                                      <  1:304033‑M2/161‑1 (MQ=255)`  
                                                                                                                                                        `aggccacacagtcacgcgctctggcgattcgctccaacgaactggctgacagtgcatccactaaagttaccgaggctgcccgtgtggcaaaccaagctcaacagctttccaaattctttgagtaatcaaacaggagaaaTCAATATGATCGTTTCTGACATCGAAGCTAACGCCCTCTTAGAGAGCGTCACTAAGTTCCACTGCGGGGTTATCTACGACTACTCCACCGCTGAGTACGTAAGCTACCGTCCGAGTGACTTCGGTGCGTATCTGGATGCGCTGGCAGCCGAGGTTGCACGAg                                                                                                                                                     >  1:59493‑M2/140‑301 (MQ=255)`  
                                                                                                                                                                  `gtcacgcgctctggcgattcgctccaacgaactggctgacagtgcatccactaaagttaccgaggctgcccgtgtggcaaaccaagctcaacagctttccaaattctttgagtaatcaaacaggagaaaTCAATATGATCGTTTCTGACATCGAAGCTAACGCCCTCTTAGAGAGCGTCACTAAGTTCCACTGCGGGGTTATCTACGACTACTCCACCGCTGAGTACGTAAGCTACCGTCCGAGTGACTTCGGTGCGTATCTGGATTCGCTGGAAGCCGAGGTTGCACGAGGCGGtcttct                                                                                                                                           >  1:187729‑M2/130‑299 (MQ=255)`  
                                                                                                                                                                  `gtcacgcgctctggcgattcgctccaacgaactggctgacagtgcatccactaaagttaccgaggctgcccgtgtggcaaaccaagctcaacagctttccaaattctttgagtaatcaaacaggagaaaTCAATATGATCGTTTCTGACATCGAAGCTAACGCCCTCTTAGAGAGCGTCACTAAGTTCCACTGCGGGGTTATCTACGACTACTCCACCGCTGAGTACGTAAGCTACCGTCCGAGTGACTTCGGTGCGTATCTGGATGCGCTGGAAGCCGAGGTTGCACGAGGCGGTCTTAt                                                                                                                                           >  1:297397‑M2/130‑301 (MQ=255)`  
                                                                                                                                                                    `cacgcgctctggcgattcgctcccacgaactggctgacagtgcatccactaaagttaccgaggctgcccgtgtggcaaaccaagctcaacagctttccaaattctttgagtaatcaaacaggagaaaTCAATATGATCGTTTCTGACATCGAAGCTAACGCCCTCTTAGAGAGCGTCACTAAGTTCCACTGCGGGGTTATCTACGACTACTCCACCGCTGAGTACGTAAGCTACCGTCCGAGTGACTTCGGTGCGTATCTGGATGCGCTGGAAGCCGAGGTTGCACGAGGCGGTCTTATtg                                                                                                                                         <  1:202371‑M2/174‑1 (MQ=255)`  
                                                                                                                                                                    `cacgcgctctggcgattcgctccaacgaactggctgacagtgcatccactaaagttaccgaggctgcccgtgtggcaaaccaagctcaacagctttccaaattctttgagtaatcaaacaggagaaaCCATTATGATCGTTTCTGACATCGAAGCTAACGCCCTCTTAGAGAGCGTCACTAAGTTCCACTGCGGGGTTATCTACGACTACTCCACCGCTGAGTACGTAAGCTACCGTCCGAGTGACTTCGGTGCGTATCTGGATGCGCTGGAAGCCGAGGTTGCACGAGGCGGTCTTattt                                                                                                                                         >  1:307189‑M2/128‑300 (MQ=255)`  
                                                                                                                                                                      `cgcgctctggcgattcgctccaacgaactggctgacagtgcatccactaaagttaccgaggctgcccgtgtggcaaaccaagctcaacagctttccaaattctttgagtaatcaaacaggagaaaTCAATATGATCGTTTCTGACATCGAAGCTAACGCCCTCTTAGAGAGCGTCACTAAGTTCCACTGCGGGGTTATCTACGACTACTCCACCGCTGAGTACGTAAGCTACCGTCCGAGTGACTTCGGTGCGTATCTGGATGCGCTGGAAGCCGAGGTTGCACGAGGCGTTCTTATtgtg                                                                                                                                       >  1:182982‑M2/126‑301 (MQ=255)`  
                                                                                                                                                                            `ctggcgattcgctccaacgaactggctgacagtgcatccactaaagttaccgaggctgcccgtgtggcaaaccaagctcaacagctttccaaattctttgagtaatcaaacaggagaaaTCAATATGATCGTTTCTGACATCGAAGCTAACGCCCTCTTAGAGAGCGTCACTAAGTTCCACTGCGGGGTTATCTACGACTACTCCACCGCTGAGTACGTAAGCTACCGTCCGAGTGACTTCGGTGCGTATCTGGATGCGCTGGAAGCCGAGGTTGCACGAGGCGGTCTTATTGTGTTCcac                                                                                                                                 <  1:194456‑M2/182‑1 (MQ=255)`  
                                                                                                                                                                                         `ccaacgaactggctgacagtgcatccactaaagttaccgaggctgcccgtgtggcaaaccaagctcaacagctttccacattctttgagtaatcaaacaggagaaaCCATTATGATCGTTTCTGACATCGAAGCTAACGCCCTCTTAGAGATCGTCACTAAGTTCCACTGCGGGGTTATCTACGACTACTCCACCGCTGAGTACGTAAGCTACCGTCCGAGTGACTTCGGTGCGTATCTGGATGCGCTGGAAGCCGAGGTTGCACGAAGCGGTCTTATTGTGTTCCACAACGGTCACAAGt                                                                                                                    >  1:161221‑M2/107‑301 (MQ=255)`  
                                                                                                                                                                                         `aaaacagactggctaacggtgcatccactaaagttaccggggctgcccgtggtgcaaaccaagctcaacagctttccaaattctttgagtaatcaaacaggagaaaTCAATCTGATCGTTTCTGACATCGAAGCTAACGCCCTCTTAGAGAGCGTCACTAAGTTCCACTGCGGTGTTATCTACGACTACTCCACCGCTGAATACGTAAGCTACCGTCCGAGTGACTTCTGTGCGTATCTGGATGCGCTGGAAGCCGAGGTTGCACGAGGCGGTCTTATTGTGTTCCACAACGGTCACAAGt                                                                                                                    <  1:154918‑M2/195‑1 (MQ=255)`  
                                                                                                                                                                                               `aactggctgactgtgcagccactaaaggtaccgaagctgcccgtgtgacaaaccaagctcaacagcttgccaaattctttgagtaataaaacaggagaaaTCAATATGATCGTTACTGACATCGAAGCTAACGCCCGCTTAGAGAGCGTCACTAAGTTCAACTGCGGGGTTATCTACGACTACTCCACCGCTGAGTACGTAAGCTACCGTCCGAGTGACTTAGGTGCGTATCTGGATGCGCTGGAAGCCGAGGTTGCACGAGGCGGTCTTATTGTGTTCCAAAACGGTCACAAGTATGACg                                                                                                              <  1:264476‑M2/201‑1 (MQ=255)`  
                                                                                                                                                                                                    `gctgacagtgcatccactaaagttaccgaggctgcccgtgtggcaaaccaagctcaacagctttccaaattctttgagtaatcaaacaggagaaaTCAATATGATCGTTTCTGACATCGAAGCTAACGCCCTCTTAGAGAGCGTCACTAAGTTCCACTGCGGGGTTATCTACGACTACTCCACCGCTGAGTACGTAAGCTACCGTCCGAGTGACTTCGGTGCGTATCTGGATGCGCTGGAAGCCGAGGTTGCCCGAGGCGGTCTTATTGTGTTCCACACCGGTCACAAGTatgacgctgtc                                                                                                         >  1:179703‑M2/96‑296 (MQ=255)`  
                                                                                                                                                                                                     `ctgacagtgcatccactaaagttaccgaggctgcccgtgtggcaaaccaagctcaacagctttccaaattctttgagtaatcaaacaggagaaaTCTATATGATCGTTTCTGACATCGAAGCTAACGCCCTCTTAGAAAGCGTCACTAAGTTCCACTGCGGGGTTATCTACGACTACTCCACCGCTGAGTACGTAAGCTACCGTCCGAGTGACTTCGGTGCGTATCTGGATGCGCTGGAAGCCGAGGTTGCACGAGGCGGTCTTATTGTGTTCCACAACGGTCACAAGTATGACGTTCCTg                                                                                                        >  1:144680‑M2/95‑301 (MQ=255)`  
                                                                                                                                                                                                        `acagtgcatccactaaagttaccgaggctgcccgtgtggcaaaccaagctcaacagctttccaaattctttgagtaatcaaacaggagaaaTCAATATGATCGTTTCTGACATCGAAGCTAACGCCCTCTTAGAGAGCGTCACTAAGTTCCACTGCGGGGTTATCTACGACTACTCCACCGCTGAGTACGTAAGCTACCGTCCGAGTGACTTCGGTGCGTATCTGGATGCGCTGGAAGCCGAGGTTGCACGAGGCGGTCTTATTGTGTTCCACAACGGTCACAAGTATGACGTTCCTGCAt                                                                                                     <  1:245360‑M2/210‑1 (MQ=255)`  
                                                                                                                                                                                                           `gtgcatccactaaagttaccgaggctgcccgtgtggcaaaccaagctcaacagctttccaaattctttgagtaatcaaacaggagaaaTCAATATGATCGTTTCTGACATCGAAGCTAACGCCCTCTTAGAGAGCGTCACTAAGTTCCACTGCGGGGTTATCTACGACTACTCCACCGCTGAGTACGTAAGCTACCGTCCGAGTGACTTCGGTGCGTATCTGGATGCGCTGGAAGCCGAGGTTGCACGAGGCggtcttattgtgttccacaactgtctcttatacacatctccgagcccac                                                                                                  >  1:219372‑M2/89‑277 (MQ=255)`  
                                                                                                                                                                                                                  `cactaaagttaccgaggctgcccgtgtggcaaaccaagctcaacagctttccaaattctttgagtaatcaaacaggagaaaCCATTATGATCGTTTCTGACATCGAAGCTAACGCCCTCTTAGAGAGCGTCACTAAGTTCCACTGCGGGGTTATCTACGACTACTCCACCGCTGAGTACGTAAGCTACCGTCCGAGTGACTTCGGTGCGTATCTGGATGCGCTGGAAGCCGAGGTTGCACGAGGCGGTCTTATTGTGTTCCACAACGGTTACAAGTATGACGTTCCTGCATTGACCAAACt                                                                                           >  1:97315‑M2/82‑301 (MQ=255)`  
                                                                                                                                                                                                                             `ccgaggctgcccgtgtggcaaaccaagctcaacagctttccaaattctttgagtaatcaaacaggagaaaCCATTATGATCGTTTCTGACATCGAAGCTAACGCCCTCTTAGAGAGCGTCACTAAGTTCCACTGCGGGGTTATCTACGACTACTCCACCGCTGAGTACGTAAGCTACCGTCCGAGTGACTTCGGTGCGTATCTGGATGCGCTGGAAGCCGAGGTTGCACGAGGCGGTCTTATTGTGTTCCACAACGGTCACAAGTATGACGTTCCTGCATTGACCAAACTGGCAAAGTTGc                                                                                >  1:275398‑M2/71‑301 (MQ=255)`  
                                                                                                                                                                                                                                `aggctgcccgtgtggcaaaccaagctcaacagctttccaaattctttgagtaatcaaacaggagaaaTCAATATGATCGTTTCTGACATCGAAGCTAACGCCCTCTTAGAGAGCGTCACTAAGTTCCACTGCGGGGTTATCTACGACTACTCCACCGCTGAGTACGTAAGCTACCGTCCGAGTGACTTCGGTGCGTATCTGGATGCGCTGGAAGCCGAGGTTGCACGAGGCGGTCTTATTGTGTTCCACAACGGTCACAAATATGACGTTCCTCCATTGACCAAACTGGCAAAGTTGCAAt                                                                             >  1:106879‑M2/68‑301 (MQ=255)`  
                                                                                                                                                                                                                                                `aaaccaagctcaacagctttccaaattctttgagtaatcaaacaggagaaaTCAATATGATCGTTTCTGACATCGAAGCTAACGCCCTCTTAGAGAGCGTCACTAAGTTCCACTGCGGGGTTATCTACGACTACTCCACCGCTGAGTACGTAAGCTACCGTCCGAGTGACTTCGGTGCGTATCTGGATGCGCTGGAAGTCGAGGTTGCACGAGGCGGTCTTATTGTGTTCCACAACGGTCACAAGTATGACGTTCCTGCATTGACCAAACTGGCAAAGTTGCAATTGAACCGAGAGTTCCa                                                             >  1:77470‑M2/52‑301 (MQ=255)`  
                                                                                                                                                                                                                                                      `agcgcaacatctttccaaattctttgggtaatcaaacaggagaaaTCATTATGATCGTTTCTGACATCGAAGCTAACGCCCTCTTAGAGAGCGTCACTAAGTTCCACTGCGGTGTTATCTACGACTACTCCACCGCTGAGTACGTAAGCTACCGTCCGAGTGACTTCGGTGCGTATCTGGATGCGCTGGAAGCCGAGGTTGCACGAGGCGGTCTTATTGTGTTCCACAACGGTCACAAGTATGACGTTCCTGCATTGACCAAACTGGCAAAGTTGCAATTGAACCGAGAGTTCCACCTTcc                                                       <  1:46814‑M2/256‑1 (MQ=255)`  
                                                                                                                                                                                                                                                         `ctcaacagctttccaaattctttgagtaatcaataggagaaaTCAATATGATCGTTTCTGACATCGAAGCTAACGCCCTCTTAGAGAGCGTCACTAAGTTCCACTGCGGGGTTATCTACGACTACTCCACCGCTGAGTACGTAAGCTACCGTCCGAGTGACTTCGGTGCGTATCTGGATGCGCTGGAAGCCGAGGTTGCACGAGGCGGTCTTATTGTGTTCCACAACGGTCACAAGTATGACGTTCCTGCATTGACCAAACTGGCAAAGTTGCAATTGAACCGAGAGTTCCACCTTCCTCg                                                    >  1:3517‑M2/43‑301 (MQ=255)`  
                                                                                                                                                                                                                                                                  `ttccaaattctttgagtaatcaaacaggagaaaTCAATATGAGCGTTTCTGACATCGAAGCTAACGCCCTCTTAGAGGGCGTCACTAAGTTCCACTGCGGGGTTATCTACGACTACTCCACCACTGAGTACGTAAGCTACCGTCCGAGTGACTTCGGTGCGTACCTGGATGAGCTGGAAGCCGAGGTTGCACGAGGCGGTCTTATTGTGTTCCGCAACGGTCACAAGTATGACGTTCCTGCATTGACCAAACTGGCAAAGTTGCAATTGAACCGAGAGTTCCACCTTCCTCGTGAGAACTg                                           <  1:141042‑M2/268‑1 (MQ=255)`  
                                                                                                                                                                                                                                                                  `ttccaaattctttgagtaatcaaacaggagaaaCCATTATGATCGTTTCTGACATCGAAGCTAACGCCCTCTTAGAGAGCGTCACTAAGTTCCACTGCGGGGTTATCTACGACTACTCCACCGCTGAGTACGTAAGCTACCGTCCGAGTGACTTCGGTGCGTATCTGGATGCGCTGGAAGCCGAGGTTGCACGAGGCGGTCTTATTGTGTTCCACAACGGTCACAAGTATGACGTTCCTGCATTGACCAAACTGGCAAAGTTGCAATTGAACCGAGAGttccaccttcctgtctcttatac                                           >  1:255754‑M2/34‑290 (MQ=255)`  
                                                                                                                                                                                                                                                                    `ccaaattctttgagtaatcaaacaggagaaaCCATTATGATCGTTTCTGACATCGAAGCTAACGCCCTCTTAGAGAGCGTCACTAAGTTCCACTGCGGGGTTATCTACGACTACTCCACCGCTGAGTACGTAAGCTACCGTCCGAGTGACTTCGGTGCGTATCTGGATGCGCTGGAAGCCGAGGTTGCACGAGGCGGTCTTATTGTGTTCCACAACGGTCACAAGTATGACGTTCCTGCATTGACCAAACTGGCAAAGTTGCAATTGAACCGAGAGTTCCACCTTCCTCGTGAGAACTGTa                                         >  1:240506‑M2/32‑301 (MQ=255)`  
                                                                                                                                                                                                                                                                        `attctttgagtaatcaaacaggagaaaTCAATATGATCGTTTCTGACATCGAAGCTAACGCCCTCTTAGAGAGCGACACTAAGTTCCACTGCGGGGTTATCTACGACTACTCCAACGCTGAGTACGAAAGCTACCGGCCGAGTGACTTCGGTGCGTATCTGGCTGCGCTGGAAACCGAGGCAGCACGAGGCGGTCTTATTGTGTTCCACAACGGTCACAAGTATGACGCTCCTACATTGACCACACTGGCAAAGTTGCAATCGAACCGAATGCTCCACCTTCCTCGTGCGAACTGACTTGa                                     >  1:93482‑M2/28‑301 (MQ=255)`  
                                                                                                                                                                                                                                                                         `ttctttgagtaatcaaacaggagaaaTCAATATGATCGTTTCTGACATCGAAGCTAACGCCCTCTTAGAGAGCGTCACTAAGTTCCACTGCGGGGTTATCTACGACTACTCCACCGCTGAGTACGTAAGCTACCGTCCGAGTGACTTCGGTGCGTATCTGGATGCGCTGGAAGCCGAGGTTGCACGAGGCGGTCTTATTGTGTTCCACAACGGTCACAAGTATGACGTTCCTGCATTGACCAAACTGGCAAAGTTGCAATTGAACCGAGAGTTCCACCTTCCTCGTGAGAACTGTATTgaa                                    >  1:294273‑M2/27‑300 (MQ=255)`  
                                                                                                                                                                                                                                                                         `ttctttgagtaatcaaacaggagaaaTCAATATGATAGTTTCTGACATCGAAGCTAACGCCTTCTTAGAGAGCGTCACTAAGTTCCACTGCGGGGTTATCTACGACTACTCCACCGCTGAGTACGTAAGCGACCGTCCGAGTGACTTCGGTGCGTATCTGGATGCGCTGGAAGCCGAGGTTGCACGAGGCGGTCTTATTGTGTTCCACAACGGTAACAAGTATGACGTTCCTGCATTGACCAAACTGGCAAAGTTGCAATTGAACCGAGAGTTCCACCTTCCTCGTGAGAACTGTATTGac                                    <  1:316300‑M2/275‑1 (MQ=255)`  
                                                                                                                                                                                                                                                                           `ctttgagtaataaaacaggagaaaACAGTATGAGCGTTCCTGACATCGAAGCTAACGCCCGCTTAGAGACCGTCACTAAGTTCCACTGAGGGGTTATCTACGACTACTCCACCGCTGAGTACGTAAGCGACCGTCCGAGTGACTTCGGTGCGTATCTGGATGCGCTGGAAGCCGAGGTTGCAAGAGGCGGTCTTATGGTGTTCCACAACGGTCACAAGTATGACGTTCCTGCATTGACCAAACTGGCAAAGTTGCAATTGAACCGAGAGTTCCACCTTCCTCGTGAGAACTGTATTGacac                                  <  1:250131‑M2/277‑1 (MQ=255)`  
                                                                                                                                                                                                                                                                                     `tcaaacaggagaaaCCATTATGATCGTTTCTGACATCGAAGCTAACGCCCTCTTAGAAAGCGTCACTAAGTTCCACTGCGGGGTTATCTACGACTACTCCACCGCTGAGTACGTAAGCTACCGTCCGAGTGACTTCGGTGCGTATCTGGATGCGCTGGAAGCCGAGGTTGCACGAGGCGGTCTTATTGTGTTCCACAACGGTCACAAGTATGACGTTCCTGCATTGACCAAACTGGCAAAGTTGCAATTGAACCGAGAGTTCCACCTTCCTCGTGAGaactgtatttacactgtatctttt                        >  1:343458‑M2/15‑291 (MQ=255)`  
                                                                                                                                                                                                                                                                                        `aacaggagaaaTCAATATGATCGTTTCTGACATCGAAGCTAACGCCCTCTTAGAGAGCGTCACTAAGTTCCACTGCGGGGTTATCTACGACTACTCCACCGCTGAGTACGTAAGCTACCGTCCGAGTGACTTCGGTGCGTATTTGGATGCGCTGGAAGCCGAGGTTGCACGAGGCGGTCTTATTGTGTTCCACAACGGTCACAAGTATGACGTTCCTGCATTGACCAAACTGGCAAAGTTGCAATTGAACCGAGAGTTCCACCTTCCTCGTGAGAACTGTATTGACACCCTTGTGTTGTCa                     <  1:33125‑M2/290‑1 (MQ=255)`  
                                                                                                                                                                                                                                                                                            `ggagaaaTCAATATGATCGTTTCTGACATCGAAGCTAACGCCCTCTTAGAGAGCGTCACTAAGTTCCACTGCGGGGTTATCTACGACTACTCCACCGCTGAGTACGTAAGCTACCGTCCGAGTGACTTCGGTGCGTATCTGGATGCGCTGGAAGCCGAGGTTGCACGAGGCGGTCTTATTGTGTTCCACAACGGTCACAAGTATGACGTTCCTGCATTGACCAAACTGGCAAAGTTGCAATTGAACCGAGAGTTCCACCTTCCTCGTGAGAACTGTATTTACACCCTTGTGTTGTCACGtt                 >  1:17020‑M2/8‑301 (MQ=255)`  
                                                                                                                                                                                                                                                                                               `gaaaTCAATATGATCGTTTCTGACATCGAAGCTAACGCCCTCTTAGAGAGCGTCACTAAGTTCCACTGCGGGGTTATCTACGACTACTCCACCGCTGAGTACGTAAGCTACCGTCCGAGTGACTTCGGTGCGTATCTGGATGCGCTGGAAGCCGAGGTTGCACGAGGCGGTCTTATTGTGTTCCACAACGGTCACAAGTATGACGTTCCTGCATTGACCAAACTGGCAAAGTTGCAATTGAACCGAGAGTTCCACCTTCCTCGTGAGAACTGTattgacacccttgtgtctgtctcttata              >  1:133837‑M2/5‑289 (MQ=255)`  
                                                                                                                                                                                                                                                                                                `aaaCCATTATGATCGTTTCTGACATCGAAGCTAACGCCCTATTAGAGAGCGTCACTAAGTTCCACTGCGGGGTTATCTACGACTACTCCACCGCTGAGTACGTAAGCTACCGTCCGAGTGACTTCGGTGCGTATCTGGATGCGCTGGAAGCCGAGGTTGCACGAGGCGGTCTTATTGTGTTCCACAACGGTCACAAGTATGACGTTCCTGCATTGACCAAACTGGCAAAGTTGCAATTGAACCGAGAGTTCCACCTTCCTCGTGAGAACTGTATTGACACCCTTGTTTTTTCACGTTTGAt             >  1:23624‑M2/4‑301 (MQ=255)`  
                                                                                                                                                                                                                                                                                                   `ccattatGATCGTTTCTGACATCGAAGCTAACGCCCTCTTAGAGAGCGTCACTAAGTTCCACTGCGGGGTTATCTACGACTACTCCACCGCTGAGTACGTAAGCTACCGTCCGAGTGACTTCGGTGCGTATCTGGATGCGCTGGAAGCCGAGGTTGCACGAGGCGGTCTTATTGTGTTCCACAACGGTCACAAGTATGACGTTCCTGCATTGACCAAACTGGCAAAGTTGCAATTGAACCGAGAGTTCCACCTTCCTCGTGAGAACTGTATTGACACCCTTGTGTTGTCACGTTTGattca          >  1:89256/5‑301 (MQ=255)`  
                                                                                                                                                                                                                                                                                                   `ccattatGATCGTTTCTGACATCGAAGCTAACGCCCTCTTAGAGAGCGTCACTAAGTTCCACTGCGGGGTTATCTACGACTACTCCACCGCTGAGTACGTAAGCTACCGTCCGAGTGACTTCGGTGCGTATCTGGATGCGCTGGAAGCCGAGGTTGCACGAGGCGGTCTTATTGTGTTCCACAACGGTCACAAGTATGACGTTCCTGCATTGACCAAACTGGCAAAGTTGCAATTGAACCGAGAGTTCCACCTTCCTCGTGAGAACTGTATTGACACCCTTGTGTTGTCACGTTTGatctt          >  1:12269/5‑298 (MQ=255)`  

                                                                                                                                                                                                                                                                                                           `|`                                                                                                                                                                                                                                                                                                              
`GACAGACTGGCTGGAAGAAACCAGAAGTTACCAAGCTGAGTCATAAGGTGTTCGATAAGGACACTATGACCCACATCAAGGCTGGTGATTGGGTTAAGGTTGACATGGGAGTTGTTGGTGGATACGGCTACGTCCGCTCAGTTAGTGGCAAATATGCACAAGTGTCATACATCACAGGTGTTACTCCACGCGGTGCAATCGTTGCCGATAAGACCAACATGATTCACACAGGTTTCTTGACAGTTGTTTCATATGAAGAGATTGTTAAGTCACGATAATCAATAGGAGAAATCAATATGATCGTTTCTGACATCGAAGCTAACGCCCTCTTAGAGAGCGTCACTAAGTTCCACTGCGGGGTTATCTACGACTACTCCACCGCTGAGTACGTAAGCTACCGTCCGAGTGACTTCGGTGCGTATCTGGATGCGCTGGAAGCCGAGGTTGCACGAGGCGGTCTTATTGTGTTCCACAACGGTCACAAGTATGACGTTCCTGCATTGACCAAACTGGCAAAGTTGCAATTGAACCGAGAGTTCCACCTTCCTCGTGAGAACTGTATTGACACCCTTGTGTTGTCACGTTTGATTCATTCCAACC  >  NC_001604.1/14057‑14656`

  

Alignment Legend

Aligned base mismatch/match (shaded by quality score): `ATCG/ATCG` `< 3 ≤` `ATCG/ATCG` `< 10 ≤` `ATCG/ATCG` `< 23 ≤` `ATCG/ATCG` `< 35 ≤` `ATCG/ATCG` `< 38 ≤` `ATCG/ATCG`

Unaligned base: `atcg`    Masked matching base: `atcg`    Alignment gap: `‑`    Deleted base: `‑`

Reads not counted as support for junction

read\_name Not counted due to insufficient overlap past the breakpoint.

read\_name Not counted due to not crossing MOB target site duplication.
